# Self-Powered p-NiO/n-ZnO Heterojunction Ultraviolet Photodetector Based on Honeycomb Nano-Mesh Structure

**DOI:** 10.3390/s24237733

**Published:** 2024-12-03

**Authors:** Nan Wang, Yang Liu, Mingyang Li, Jieting Zhao, Xiaoqi Zhang, Dayong Jiang

**Affiliations:** 1 School of Engineering, Changchun Normal University, Changchun 130032, China; zhaojieting@ccsfu.edu.cn (J.Z.); zhangxq@ccsfu.edu.cn (X.Z.); 2 Engineering Research Center of Jilin Province Rare Metal Deep Processing, Changchun 130022, China; 3 Engineering Research Center of Jilin Province Intelligent Manufacturing Equipment R&D and Testing, Changchun 130022, China; 4 College of Electromechanical, Changchun Polytechnic, Changchun 130033, China; 2015200024@mails.cust.edu.cn; 5 School of Materials Science and Engineering, Changchun University of Science and Technology, Changchun 130022, China; 2021200151@mails.cust.edu.cn (M.L.); jiangdy@cust.edu.cn (D.J.)

**Keywords:** ZnO honeycomb nano-mesh, light-trapping effect, heterojunction, ultraviolet photodetector

## Abstract

Ultraviolet (UV) photodetectors (PDs) are characterized by wide wavelength selectivity and strong anti-interference capability. The focus of research is not only limited to the adjustment of the structure composition, but it also delves deeper into its working mechanism and performance optimization. In this study, a heterojunction self-powered photodetector with a unique honeycomb structure was successfully constructed by combining the advantages of two semiconductor materials, zinc oxide (ZnO) and nickel oxide (NiO), using magnetron sputtering and hydrothermal synthesis. The detector has high responsivity, high detectivity and favorable spectral selectivity under UV irradiation. The nearly 10-fold increase in responsivity and detectivity of the detector with the introduction of the honeycomb structure under zero-bias conditions is attributed to the macroporous structure of the ZnO honeycomb nano-mesh, which increases the surface active sites and facilitates the enhancement of light trapping. This study provides significant value to the field of UV detection by improving detector performance through structural optimization.

## 1. Introduction

PDs are devices that convert an optical signal into an electrical signal and play a vital role in military, space, biomedical and environmental monitoring [1,2,3]. UV light has strong penetrating power and high monochromaticity, which makes UV PDs have the advantages of high sensitivity, high selectivity and low background noise [4,5]. ZnO is a wide-bandgap semiconductor material which is sensitive to UV light and is suitable for the detection of radiation in the UV region [6,7,8]. ZnO has better electron transport properties and can be used as the electron transport layer of PDs to improve the mobility of carriers [9,10,11]. Research on ZnO UV PDs aims to improve their sensitivity and anti-interference capability [12,13]. In complex environments, how to allow ZnO UV PDs to capture weak UV signals accurately and stably is the focus of current research [14,15].

Structural design challenges are among the bottlenecks in ZnO UV PDs [16,17,18]. The heterostructure can effectively separate the photogenerated electron–hole pairs and reduce the recombination rate, thus improving photoelectric conversion efficiency [19,20,21]. As a P-type semiconductor, NiO has a high light absorption coefficient [22,23]. ZnO/NiO heterostructure PDs have broad spectral selectivity and responsiveness in the UV band. At the same time, the presence of a built-in electric field makes the device self-powered and able to work without external power supply [24,25]. However, the ZnO/NiO heterostructure, during the fabrication process, is prone to the introduction of defect states at the interface due to lattice mismatch and differences in chemical composition [26,27]. These defect states may trap and recombine photogenerated carriers, leading to a decrease in carrier transport efficiency, which in turn affects the device’s responsivity and sensitivity [28,29]. Nanomaterials are subject to the surface effect, the small-size effect, the quantum size effect and the macroscopic quantum tunneling effect [30,31,32]. For nanomaterial PDs, one-dimensional nanowire structures have been the subject of extensive research [33,34,35]. In fact, the honeycomb nano-mesh structure is also an excellent nanoscopic configuration. The honeycomb nano-mesh provides high specific surface area and more active sites for carrier generation. Meanwhile, the unique pores of the honeycomb nano-mesh structure can be used as a light-trapping mechanism to reduce light loss and improve light absorption efficiency.

In this study, a ZnO NM/ZnO/NiO self-powered PD was successfully prepared by controlling the growth parameters. The experimental results show that efficient photoelectric conversion is realized due to energy band matching and the built-in electric field effect between the two semiconductor materials. Meanwhile, the large pore structure of the ZnO honeycomb nano-mesh increases the surface active sites and promotes the enhancement of light trapping. This work provides important material design ideas and preparation methods for the development of novel semiconductor heterostructures, which is significant for the development of efficient PDs.

## 2. Materials and Methods

Materials: NiO target (99.9%), ZnO target (99.99%), Al target (99.999%), zinc acetate (Zn(CH_3_COO)^2^·2H_2_O; ≥99.0%), hexamethylenetetramine (HTMA; ≥99.0%), acetone (C_3_H_6_O; ≥99.5%) and ethanol (C_2_H_6_O; ≥99.0%).

Fabrication of ZnO NM/ZnO/NiO PD: An ITO quartz substrate (2.0 cm × 1.0 cm) was cleaned in acetone, ethanol and deionized water for 10 min each and dried with nitrogen. NiO films were obtained by RF magnetron sputtering under the operating conditions of pressure of 1 Pa, power of 90 W and O_2_/Ar ratio of 9:1. ZnO films were deposited on the NiO films by magnetron sputtering under the operating conditions of pressure of 0.6 Pa, power of 150 W and O_2_/Ar ratio of 10:40. The prepared ZnO/NiO film substrate was inverted on a support and placed in a PTFE reactor. The reactor was prepared with 2 mmol/L Zn(CH_3_COO)^2^·2H_2_O and 2 mmol/L HMTA reaction solution. After 12 h of reaction at 90 °C, the honeycomb nano-mesh structure was formed. The device was placed into the evaporation chamber with a metal mask plate, and a dock evaporation boat was used as the evaporation source to hold the Al grains. The evaporation duration was 10 min, and the substrate speed was 5 r/min. The final Al top electrode was obtained with a size of 1.0 cm × 1.0 cm. For comparison, ZnO/NiO PD and NiO PD were fabricated by using the above method.

Characterization: X-ray diffraction (XRD) curves were obtained by using a D/MAX2550 X-ray powder diffractometer (Rigaku, Tokyo, Japan). The absorption intensities of the PDs were characterized by using a PU-1901 dual-beam UV-visible spectrophotometer (Persee, Beijing, China). A scanning electron microscope (SEM) was used to characterize the morphology of the honeycomb nano-mesh and thin films (Hitachi, Tokyo, Japan). The responsivity of the PDs was measured by using a DR800-CUST (Zolix, Beijing, China). The current–voltage characteristics of the PDs were analyzed by using Keithley 2400 (Tektronix, OH, USA).

## 3. Results

**Figure 1:**
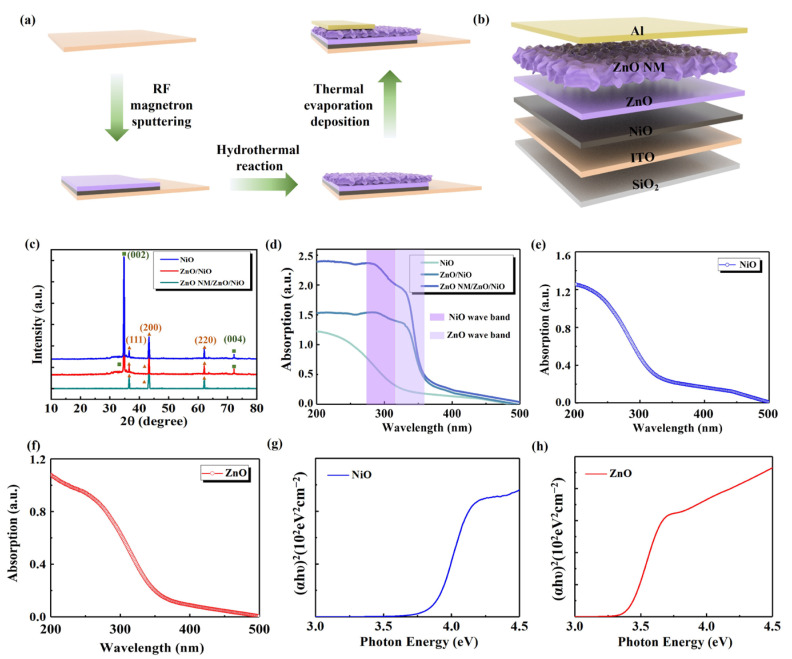
(**a**) Preparation process of ZnO NM/ZnO/NiO PD. (**b**) Structure diagram of ZnO NM/ZnO/NiO PD. (**c**) XRD patterns of devices. (**d**) UV absorption spectra of devices. (**e**) UV absorption spectra of NiO. (**f**) UV absorption spectra of ZnO. (**g**) Curve of (αhυ)^2^ vs hυ for NiO. (**h**) Curve of (αhγ)^2^ vs. hγ for ZnO.

Figure 1a shows the process of fabricating the detector. Figure 1b shows the structure diagram of the detector. From Figure 1c, the characteristic peaks of NiO were found at (111), (200) and (220) corresponding to 31.5°, 34.6° and 62.1°, respectively. The characteristic peaks of ZnO corresponded to (002) and (004) crystal diffraction planes, which were 34.8° and 72.1°, respectively. In the absorption spectroscopy test, the light absorption characteristics of the devices in the wavelength range of 200 to 500 nm were investigated (Figure 1d–f). The results show that the absorption intensities of the three different PDs show an increasing trend with the shortening of the wavelength, which is especially significant in the UV region. This indicates that the device has high sensitivity to light at these wavelengths. Specifically, since the forbidden bandwidth of NiO is wider than that of ZnO, the absorption cut-off for NiO is located at a shorter wavelength, around 330 nm, while that for ZnO is located at around 365 nm. Notably, the absorption of ZnO NM/ZnO/NiO PD is significantly higher than that of the pure thin-film PDs, which suggests that the introduction of the honeycomb nano-mesh structure significantly enhances the absorption of the devices in the UV region and results in a steeper absorption cut-off edge. In Figure 1g,h, the bandgap of the film is determined by fitting a single function to the curve of the linear part of the absorption spectrum and extrapolating the fitted straight line to the horizontal *x*-axis, i.e., (αhυ)^2^ = 0 [36,37]. By this method, the forbidden bandwidths of the NiO and ZnO were calculated to be about 3.76 eV and 3.40 eV, respectively, which further confirms the difference in energy bands between NiO and ZnO.

**Figure 2:**
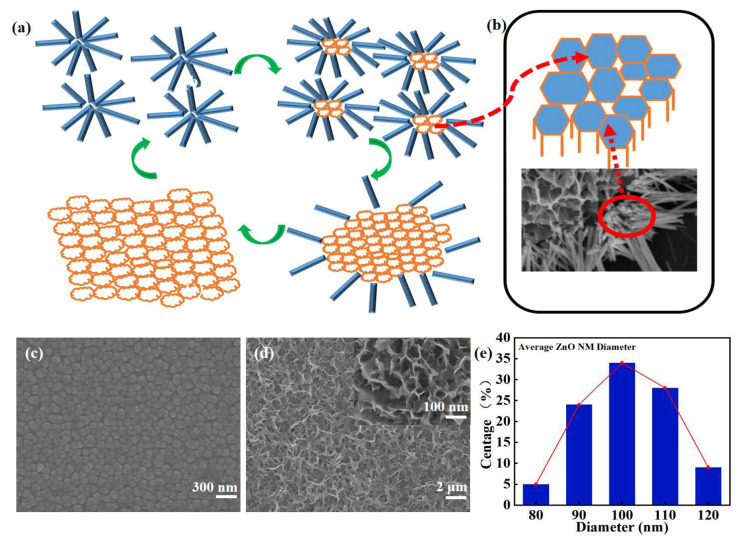
(**a**) Schematic diagram of growth of ZnO honeycomb nano-mesh materials. (**b**) Diagrams of nucleation of nanowire clusters and accompanying pore structure. SEM of (**c**) ZnO thin films and (**d**) honeycomb nano-mesh materials. (**e**) Diameter distribution of honeycomb nanopores.

Figure 2a details the steps of the formation of the ZnO honeycomb nano-mesh nanostructures during a 12 h hydrothermal reaction. The self-assembly and transformation mechanisms are described below. Based on the morphological changes, several key growth stages can be identified: (I) the formation of nanowires and oriented aggregation to form clusters, (II) nucleation process inside the clusters, (III) further growth of the nuclei and (IV) the formation of the honeycomb mesh layer. The nucleation process of the ZnO materials in solution was initiated at a certain time as the reaction time increased [38,39]. This process is based on a pre-prepared thin-film layer of ZnO, which provides the basis for the nucleation and subsequent growth of ZnO. In Figure 2b, the nucleation of nanowires clusters and the accompanying pore structure can be observed. As the reaction proceeds, the cores continuously absorb the surrounding nanowires. Upon completion of the reaction, the nanowires are absent, and a honeycomb pore-like nanostructure is formed. In Figure 2c, it can be observed that the surface grains of the ZnO thin films show a tight and consistent growth pattern, with the size of the grains being about 100 nm. This indicates that the ZnO layer deposited by the RF magnetron sputtering technique has a preferred crystal orientation which significantly influences the growth direction and morphology of the ZnO nanomaterials. Further, Figure 2d reveals the morphological features of ZnO honeycomb nano-mesh after the reaction is complete. These nanomaterials have a pore size of approximately 100 nm and exhibit a large and uniform pore structure. In addition, by using Image J software (1.8.0), a distribution map of the variation in nanopore diameters in the range of 1 × 1 µm was obtained (Figure 2e). According to this distribution plot, the average diameter range of the ZnO honeycomb pores is around 100 nm. ZnO has a hexagonal fibrillated zincite structure, and the ZnO films provide specific crystal orientation and uniform nucleation sites for the growth of nanowires [40]. These sites help the nanowires to nucleate and grow uniformly in the initial stage, thus reducing the possibility of random nucleation and uneven growth [38].

**Figure 3:**
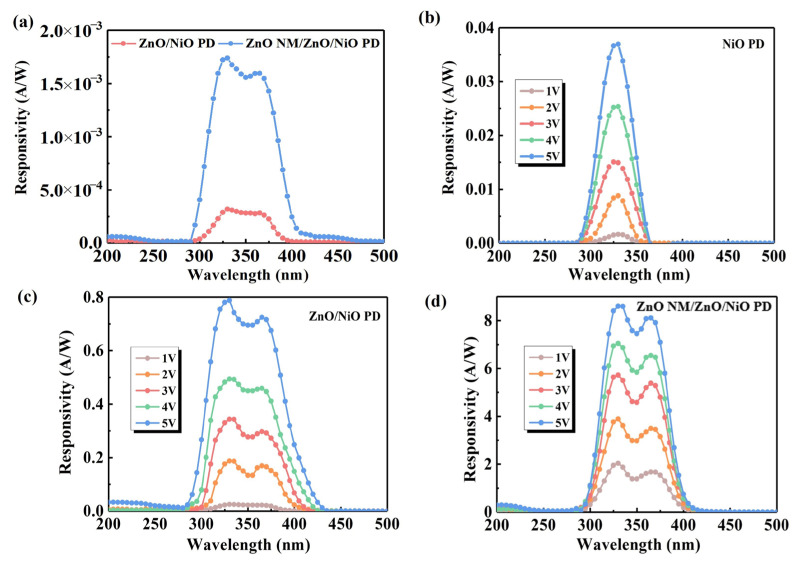
Responsivity spectrum of (**a**) ZnO/NiO PD and ZnO NM/ZnO/NiO at 0 V bias voltage, (**b**) NiO PD at 1–5 V bias voltage, (**c**) ZnO/NiO PD at 1–5 V bias voltage and (**d**) ZnO NM/ZnO/NiO PD at 1–5 V bias voltage.

Without external bias, both ZnO/NiO and ZnO NW/ZnO/NiO devices exhibit sensitivity to near-ultraviolet light (Figure 3a), suggesting that the detectors have the ability to be self-powered. The self-powering property originates from the energy band bending that occurs when p-NiO is in contact with n-ZnO, which creates a built-in electric field at the contact interface. The responsivity of three sets of PDs was measured at bias voltages ranging from 1 to 5 V in a wavelength range consistent with the range of the absorption curves from 200 to 500 nm. All detectors exhibited significant wavelength selectivity and strong response to UV light at lower radiation intensities. Specifically, the data in Figure 3b show that the conventional thin-film NiO PD has relatively low responsivity of about 0.04 A/W. In contrast, the responsivity curves in Figure 3c reveal two distinct peaks that correspond to the absorption cut-off edges of the p-NiO and n-ZnO films shown in Figure 1d at 330 nm and 365 nm, respectively. Further, the responsivity of the ZnO NM/ZnO/NiO PD is significantly improved compared with the thin-film detector, as shown in Figure 3d. The responsivity of the detector at 330 nm is as high as 8.60 A/W at 5 V. The improved responsivity property is mainly attributed to the light-trapping effect of the honeycomb nano-mesh structure and the large void structure that significantly increases the specific surface area of the materials.

**Figure 4:**
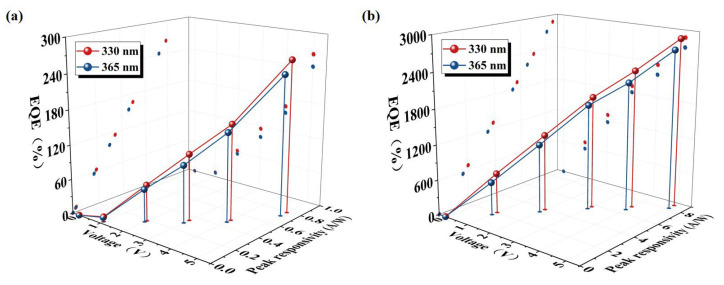
Group plots of peak responsivity and *EQE* for (**a**) ZnO/NiO PD and (**b**) ZnO NM/ZnO/NiO PD.

External quantum efficiency (*EQE*) is a key measure of PD photovoltaic conversion efficiency. *EQE* is calculated by the following formula [41]:

*EQE* = (*R_λ_* × *h* × *c*)/(*q* × *λ*) × 100% (1)

here, *R_λ_* refers to the responsivity at a specific wavelength *λ*, *h* denotes the Planck constant, *c* is the speed of light, *q* stands for the charge of the electrons (1.60 × 10^−19^ C), and *λ* represents the wavelength of incident light. According to Equation (1), it can be deduced that there is a proportional relationship between *EQE* and responsivity, which means that an increase in responsivity directly leads to an increase in *EQE*. The relationship between peak responsivity and *EQE* for two different wavelengths of light (330 nm and 365 nm) as the voltage is varied is demonstrated in Figure 4. The responsivity of the ZnO/NiO PD at the 330 nm wavelength under the unbiased (0 V) condition is 0.29 mA/W, which corresponds to an *EQE* of 0.09%. However, the ZnO NM/ZnO/NiO PD performs even better, with a responsivity of 1.74 mA/W and an improved *EQE* of 0.58%. These data clearly indicate that the introduction of the ZnO honeycomb nano-mesh structure significantly enhances the photocurrent and thus the sensitivity of the detector to UV light. It was further observed that the responsivity and *EQE* of the PD show an increasing trend with the increase in bias voltage. In particular, at lower bias voltages, the combination of the ZnO honeycomb nano-mesh with the thin film can induce significant internal gain, which further enhances detector performance.

**Figure 5:**
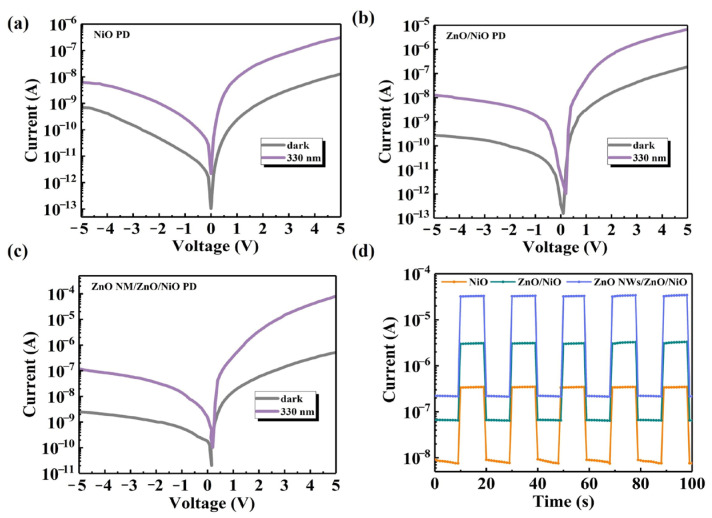
The *I*–*V* characteristics in the dark and at 330 nm of the (**a**) NiO PD, (**b**) ZnO/NiO PD and (**c**) ZnO NM/ZnO/NiO PD. (**d**) The switching stability of the PDs demonstrated at 5 V.

For exploring the current–voltage (*I*–*V*) characteristics of the PDs, experiments were conducted both in a dark environment and under 330 nm UV irradiation. The dark-current variations of the three groups of UV PDs in Figure 5a–c clearly demonstrate that all the PDs exhibit good rectification behavior, which indicates the stability of their electrical properties. It is noteworthy that all samples exhibit low dark-current levels. The nonlinear relationship between current and voltage in Figure 5b,c demonstrates the characteristics of high unidirectional conductivity and low leakage current of these PDs, which further confirms the formation of an effective heterostructure between the ZnO and NiO films. The photocurrent variation results show that the photocurrents of ZnO NM/ZnO/NiO PD and ZnO/NiO PD are significantly enhanced after the formation of heterojunctions. The *I*–*V* characteristic curves indicate that the PDs based on spectrally selective heterojunctions, both ZnO/NiO PDs and ZnO NWs/ZnO/NiO PDs, are capable of achieving stable photoresponses and that they can be operated without the need of an external power source for normal operation. The photoresponse cycles under 365 nm illumination are given in Figure 5d, showing their switching stability when varying the incident light.

**Figure 6:**
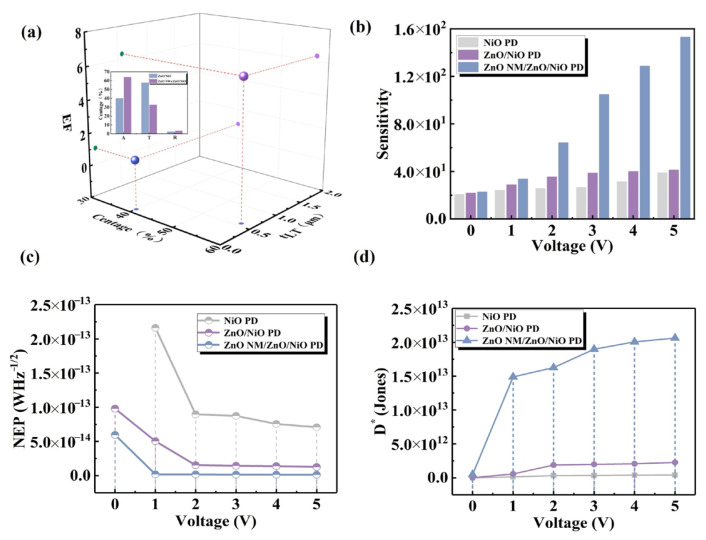
(**a**) Diagram of *t_LT_* vs. EF of PDs, with inset showing absorption, reflectance and transmission diagrams of PDs. Performance metrics for PDs at 0–5 V bias voltage as (**b**) sensitivity, (**c**) *NEP* and (**d**) D*.

Figure 6a presents the *t_LT_* and *EF* values for the two self-powered PDs. The inset graph displays the absorption, transmission and reflectance values for both PDs. The high *t_LT_* and *EF* values for the ZnO NWs/ZnO/NiO PD demonstrate that the NWs serve as highly efficient scattering centers, effectively coupling most of the incident light into the film at various angles. This geometry of the NWs significantly improves light-trapping efficiency. In order to more clearly understand and evaluate the performance of the detectors, three key parameters, sensitivity, detection rate (D*) and noise equivalent power (*NEP*), were calculated for the PDs (Figure 6b–d). Sensitivity usually refers to the degree of response of the detector output signal with respect to the input signal and is defined by calculating the ratio of (*I_light_*−*I_dark_*)/*I_dark_* [42]. As shown in Figure 6b, the ZnO NM/ZnO/NiO PD exhibits higher sensitivity, indicating that the detector is more sensitive to the response of light signals. *NEP* is a direct measure of the detector’s sensitivity to weak signals (Figure 6c). *NEP* is calculated by the following equation [43]:

(2) $$\mathrm{NEP}={\left(2qI_{\mathrm{dark}}\right)}^{1/2}/R$$

The *NEP* value of the PDs decreases after the introduction of the ZnO honeycomb nano-mesh structure. Reducing the *NEP* improves the performance of the detector under low-light conditions, making it more suitable for applications that require high-sensitivity detection. Detectivity (D*) is a comprehensive performance parameter that combines the responsivity, bandwidth and noise characteristics of PDs (Figure 6d). D* is calculated as follows [44]:

(3) $$D^{*}=RA^{1/2}/{(2qI_{\mathrm{dark}})}^{1/2}$$

where *A* is the effective area (0.392 cm^2^). The one order of magnitude improvement in D* of the ZnO NM/ZnO/NiO PD compared with the thin-film PDs implies that the detector’s ability to detect signals at lower optical power levels is enhanced. The structure of the ZnO honeycomb nano-mesh significantly reduces the reflectivity of the light and reduces the surface escape effect, which in turn enhances photon utilization efficiency.

**Figure 7:**
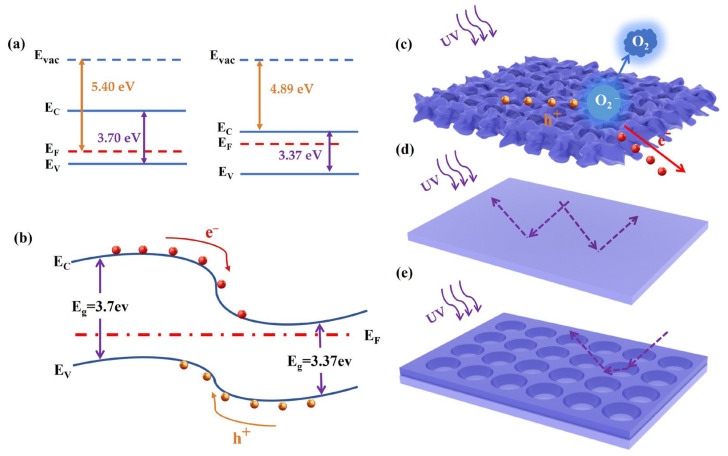
(**a**) Energy band diagram representing isolated NiO and ZnO. (**b**) Schematic diagram of charge distribution and direction in space of ZnO/NiO built-in electric field. (**c**) Distribution of electrons and holes on surface of honeycomb nano-mesh during illumination. Light path diagrams of light-emitting diodes with (**d**) ZnO/NiO PD and (**e**) ZnO NM/ZnO/NiO PD.

The energy band arrangements for standalone ZnO and NiO are depicted in Figure 7a. When ZnO and NiO are in contact to form a heterojunction, due to the different Fermi energy levels of the two materials, electrons flow from the material with a high Fermi energy level (NiO) to the material with a low Fermi energy level (ZnO) until the Fermi energy levels of the two reach the equilibrium (Figure 7b). During this process, a built-in electric field is formed at the heterojunction interface, which is directed from ZnO to NiO. Energy band bending helps the separation of electrons and holes at the heterojunction interface. Under the effect of the built-in electric field, the electrons move toward the ZnO side, while the holes move toward the NiO side [45]. The separation facilitates the fast transport and collection of photogenerated electron–hole pairs, which improves the performance of the heterojunction device [46]. Under normal environmental conditions, oxygen molecules attach to the surface of the nano-mesh and seize free electrons from them, following the reaction [O_2_+e^−^→O^2−^] [47]. This process leads to the formation of a depletion layer with low conductivity near the honeycomb nano-mesh surface. When exposed to UV light, the device produces electron–hole pairs. The holes travel to the surface to detach the adsorbed oxygen through the reaction [h^+^+O^2−^→O_2_], which decreases the thickness of the depletion barrier and boosts the concentration of free carriers (Figure 7c). Consequently, the photocurrent surges significantly upon UV illumination [48]. Figure 7d,e show the schematic structure of thin film and honeycomb nano-mesh when UV light is incident. As shown in Figure 7d, the direct reflection and refraction of the light incident on the smooth film surface causes a large amount of light loss, which makes the film structure absorb less light. The phenomenon is analyzed from the trend of the absorption spectrum in Figure 1d. As shown in Figure 7e, the large pores of the honeycomb nano-mesh allow the incident light to be reflected, refracted and scattered at different angles within it. This effect leads to a longer propagation path of the light inside the material, increasing the interaction time between the light and the material, thus improving the efficiency of light absorption or light scattering.

## 4. Conclusions

In this study, a high-performance device was successfully designed and prepared by focusing on a ZnO/NiO PD with honeycomb network structure based on the light-trapping effect. The detector utilizes the light-trapping effect of the heterostructure and nanostructure to significantly improve the absorption of UV light and achieve excellent photoelectric performance. The ZnO NM/ZnO/NiO PD exhibits significant responsivity at 330 nm and 365 nm, with responsivity values of 8.6 A/W and 8.2 A/W, respectively. Specifically, the ZnO honeycomb nano-mesh layer has a large interfacial state density and a surface pit structure, which are properties that can effectively improve the light reflection on the surface of the thin film, thus facilitating light trapping and absorption. This study also provides new ideas and methods for the application of the light-trapping effect in the field of optoelectronic detection and makes a positive contribution to the research and development of related fields.

## Data Availability

The data presented in this study are available upon request from the corresponding author. The data are not publicly available due to their large dimensions.
